# Evolution of land plant genes encoding L-Ala-D/L-Glu epimerases (AEEs) via horizontal gene transfer and positive selection

**DOI:** 10.1186/1471-2229-13-34

**Published:** 2013-03-01

**Authors:** Zefeng Yang, Yifan Wang, Yong Zhou, Qingsong Gao, Enying Zhang, Lei Zhu, Yunyun Hu, Chenwu Xu

**Affiliations:** 1 Jiangsu Provincial Key Laboratory of Crop Genetics and Physiology; Key Laboratory of Plant Functional Genomics of Ministry of Education, College of Agriculture, Yangzhou University, Yangzhou, 225009, China

**Keywords:** Land plants, L-Ala-D/L-Glu epimerase, Horizontal gene transfer, Bacteria

## Abstract

**Background:**

The L-Ala-D/L-Glu epimerases (AEEs), a subgroup of the enolase superfamily, catalyze the epimerization of L-Ala-D/L-Glu and other dipeptides in bacteria and contribute to the metabolism of the murein peptide of peptidoglycan. Although lacking in peptidoglycan, land plants possess *AEE* genes that show high similarity to those in bacteria.

**Results:**

Similarity searches revealed that the *AEE* gene is ubiquitous in land plants, from bryophytas to angiosperms. However, other eukaryotes, including green and red algae, do not contain genes encoding proteins with an L-Ala-D/L-Glu_epimerase domain. Homologs of land plant *AEE* genes were found to only be present in prokaryotes, especially in bacteria. Phylogenetic analysis revealed that the land plant *AEE* genes formed a monophyletic group with some bacterial homologs. In addition, land plant AEE proteins showed the highest similarity with these bacterial homologs and shared motifs only conserved in land plant and these bacterial AEEs. Integrated information on the taxonomic distribution, phylogenetic relationships and sequence similarity of the AEE proteins revealed that the land plant *AEE* genes were acquired from bacteria through an ancient horizontal gene transfer (HGT) event. Further evidence revealed that land plant *AEE* genes had undergone positive selection and formed the main characteristics of exon/intron structures through gaining some introns during the initially evolutionary period in the ancestor of land plants.

**Conclusions:**

The results of this study clearly demonstrated that the ancestor of land plants acquired an *AEE* gene from bacteria via an ancient HGT event. Other findings illustrated that adaptive evolution through positive selection has contributed to the functional adaptation and fixation of this gene in land plants.

## Background

Horizontal gene transfer (HGT), also known as lateral gene transfer (LGT), refers to the transfer of genetic material between organisms that are reproductively isolated [1,2]. HGT plays important roles in accelerating the evolution of the acceptor lineages because it can considerably expand the gene pool beyond species barriers. It is believed that HGT is one of the major forces driving the evolution of prokaryotes, leading to the acquisition or modification of certain adaptive traits, such as antibiotic resistance, virulence, and photosynthesis [3]. For example, a genome-wide analysis revealed that 755 out of 4,288 genes have been transferred to the *Escherichia coli* genome, and at least 234 HGT events contributed to the origin of these transferred genes [4].

The frequency of HGT into eukaryotic genomes is possibly lower than in prokaryotes, but HGT has also been an important force in the evolution of eukaryotes [5]. Genome-wide identification revealed that 7.6% of the secreted proteome of *Phytophthora ramorum* has been acquired from fungi via HGT, suggesting that oomycetes became successful plant parasites through multiple acquisitions of genes from fungi [6]. Single-celled organisms have been found to be the dominant agent for genetic transfer, and many microbial eukaryotes and plant mitochondria provide rich in examples of HGT [7]. Gene recruitment has been thought to be difficult in multi-cellular eukaryotes. However, recent investigations in plants, fungi and animals have refuted this conjecture [8,9]. The classical example of plant HGT is that the ct-DNA sequences in some tobacco nuclear genomes were probably horizontally acquired from *Agrobacterium rhizogenes* during ancient infections [10]. In addition, evidence has also showed that land plants can recruit genes from species with distinct relationships, such as fungi, bacteria, and other plant species [11-15]. Although land plant genes acquired through HGT are quite rare, they might play critical roles in adaptation to environments. For example, some anciently employed genes have been found to be involved in many plant-specific activities, including xylem formation, plant defense, nitrogen recycling and the biosynthesis of starch, polyamines, hormones and glutathione [14].

L-Ala-D/L-Glu epimerase (AEE) belongs to the enolase superfamily and catalyzes the epimerization of L-Ala-D/L-Glu and other dipeptides [16]. Studies examining *AEEs* in *E. coli* and *Bacillus subtilis* indicated a probable role in the metabolism of the murein peptide of peptidoglycan, of which L-Ala-D-Glu is a component [16,17]. However, it has been shown that the AEE family contains members from plants and archaea that lack peptidoglycan, suggesting that the proteins of this family might have other functions [18]. A recent investigation in *Thermotoga martima*, a species of thermotoga bacteria, resulted in the assignment of epimerase activity for L-Ala-D/L-Phe, L-Ala-D/L-Tyr, and L-Ala-D/L-His to one member of the AEE family [18]. It has also been noted that the genomes of *Oryza* and *Arabidopsis* possess genes encoding AEEs [19]. However, the AEE member found in *Arabidopsis* has long been annotated as a protein of the cytochrome P450 superfamily and was looked at as a pseudogene until it was found to be expressed [20]. Because the *Arabidopsis* AEE shows similarity with bacterial TfdD, an enzyme in the degradation pathway for chlorinated aromatics, it is assumed to have the potential to degrade aromatic compounds when the bacterial *TfdC* gene is introduced to the plant [21].

The ubiquity of the *AEE* gene in land plants suggests that its functions could include a wide range of selectivity, although its actual function remains unclear. The origin of this gene in land plants remains unknown at present. The increased availability of AEE sequences in public databases allows us to explore the functional diversity from a phylogenetic perspective within the AEE family in land plants. Here, we examined the evolutionary relationship of the land plant *AEE* genes and their homologs in cellular organisms. Our bioinformatic analyses revealed that land plant *AEE* genes originated from an ancient HGT event, and the putative donor was bacteria. Further evidence showed that positive selection followed by purifying selection has contributed to the evolution of this gene in land plants.

## Results

### *AEE* genes in land plants

**Figure 1:**
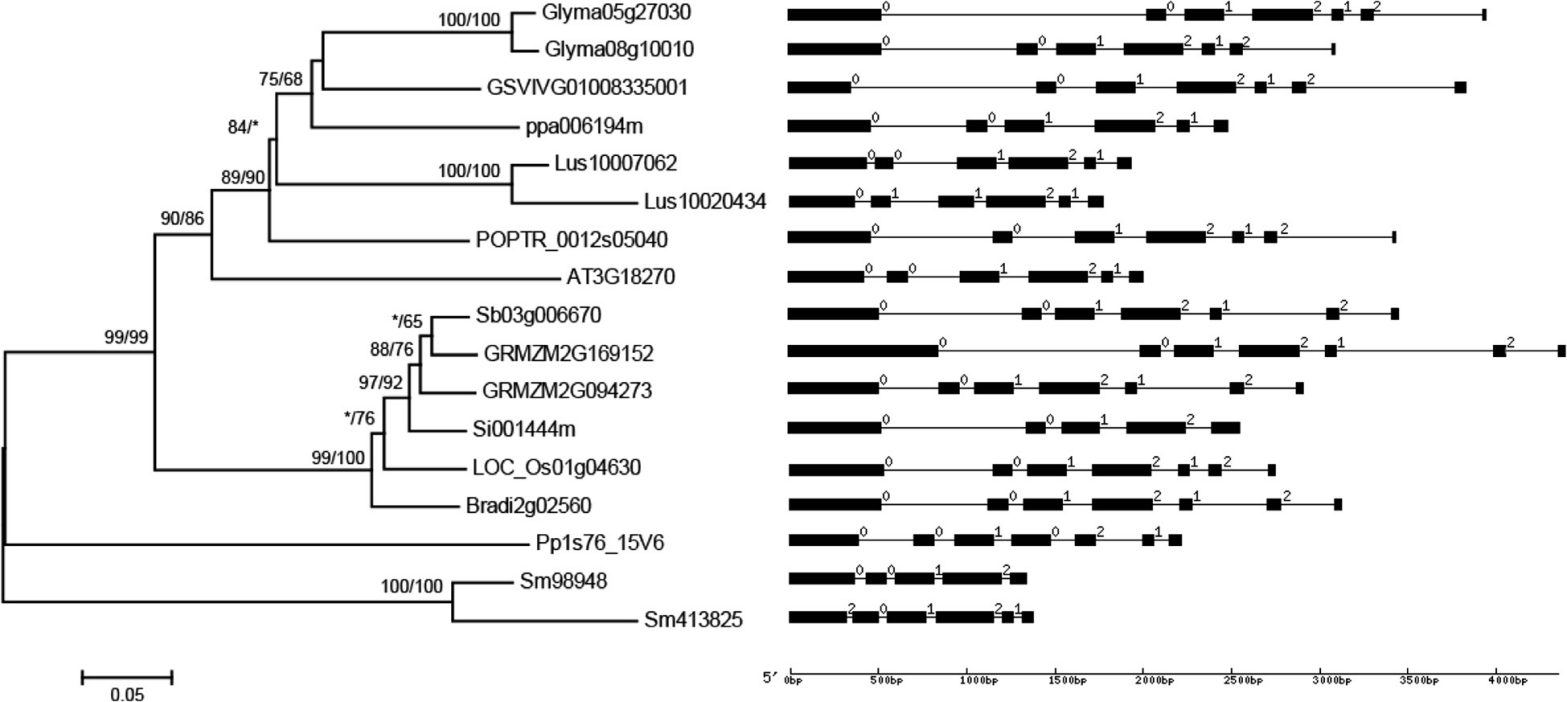
**Phylogenetic tree of land plant *AEE *genes and their exon/intron structures.** The numbers above the branches provide the bootstrap values for maximum likelihood and distance analyses, respectively. Asterisks indicate values lower than 50%. Exons are indicated by boxes, while introns are indicated by lines. The number above an intron indicates the phase.

BLAST searches revealed *AEE* genes in various land plants, including bryophytas, lycophytes, gymnosperms, monocots and dicots; all of the sequenced land plant genomes were found to contain at least one *AEE* gene. Although no whole-genome sequences have yet been reported for gymnosperms, several ESTs from *Picea sitchensis*, *P. glauca*, *Pinus taeda* and *P. contorta* showed high similarity with *AEE* genes. To explore the origin and evolution of the land plants *AEE* genes, we characterized *AEE* genes from species representing the main lineages of land plants, including the bryophyta *Physcomitrella patens*, the lycophyte *Selaginella moellendorffii*, and 5 monocot and 6 dicot angiosperms (Table 1). Among the tested land plant genomes, four (*Linum usitatissimum*, *Glycine max*, *Zea mays* and *S. moellendorffii*) contained two *AEE* genes, and all other genomes contained only one. In the phylogeny (Figure 1), all of the paralogous genes were found to be located at the termini of branches, illustrating that these paralogs were formed through recent duplication events. We also found that three paralogs in angiosperms were results of segmental duplication because there were highly conserved genes within the flanking regions of these three pairs of paralogous *AEE* genes. However, the locations in neighboring genomic regions revealed that the paralogous pair in *S. moellendorffii* was the result of tandem duplication. BLAST searches showed that all of the *AEE* genes in land plants exhibited at least one significant EST hit in NCBI, except for two genes from *L. usitatissimum*. The land plant *AEE* genes generally contained 4–6 introns in their coding regions. Although intron gain/loss was also found in some species, the structure of these *AEE* genes showed highly similarity (Figure 1), illustrating that the main characteristics of the gene structure of this family were formed in the common ancestor of land plants.

**Table 1:** **List of *AEE *genes in 13 representative land plant genomes**

**Lineage**	**Species**	**Locus**^**a**^	**Length (aa)**	**Intron**	**Chr/scaffold**	**Location**	**EST hits**
Dicot	*Arabidopsis thaliana*	AT3G18270	410	5	3	6261905 - 6264309	11
	*Populus trichocarpa*	POPTR_0012s05040	421	6	scaffold_12	4532099 - 4536153	5
	*Glycine max*	Glyma05g27030	443	6	5	32914724 - 32919062	19
	*Glycine max*	Glyma08g10010	443	6	8	7188312 - 7191927	21
	*Prunus persica*	ppa006194m	423	5	scaffold_5	17393161 - 17395652	1
	*Vitis vinifera*	GSVIVG01008335001	401	6	17	3148656 - 3153072	4
	*Linum usitatissimum*	Lus10020434	383	5	scaffold44	148949 - 150726	0
	*Linum usitatissimum*	Lus10007062	408	5	scaffold772	42573 - 44511	0
Monocot	*Sorghum bicolor*	Sb03g006670	439	6	3	6866003 - 6869858	8
	*Setaria italica*	Si001444m	447	4	scaffold_5	8094542 - 8098228	1
	*Oryza sativa*	LOC_Os01g04630	446	6	1	2074753 - 2077903	19
	*Zea mays*	GRMZM2G169152	552	6	3	19939526 - 19944315	22
	*Zea mays*	GRMZM2G094273	440	6	8	13336541 - 13339982	1
	*Brachypodium distachyon*	Bradi2g02560	442	6	2	1705605 - 1709251	2
Lycophyte	*Selaginella moellendorffii*	413825	378	5	scaffold_21	1839471 - 1840852	2
	*Selaginella moellendorffii*	98948	374	4	scaffold_21	1851300 - 1852645	2
Bryophyta	*Physcomitrella patens*	Pp1s76_15V6	397	6	scaffold_76	98135 - 100510	8

^a^ The locus and the sequence information for each gene in this table were obtained from Phytozome v9.0 (http://www.phytozome.net/).

### The origin of land plant *AEE* genes

There is no doubt that land plants originated from green algae and that most of the genes in the genomes of land plants were vertically inherited from their common ancestor [22,23]. We searched the *nr* and EST databases of NCBI and available eukaryotic genome databases for homologs of land plant *AEE* genes. To our surprise, the results indicated that there was no homolog in any other eukaryote, including in the genomes of green and red algae. Blast results also revealed that homologs of land plant AEE proteins only existed in prokaryotes, mainly in bacteria. The taxonomic distribution of the *AEE* genes suggested that their emergence in land plants might have been a result of horizontal gene transfer (HGT) from a prokaryote, and the universality of their distribution in bacteria also suggested that this gene first emerged in bacteria.

**Figure 2:**
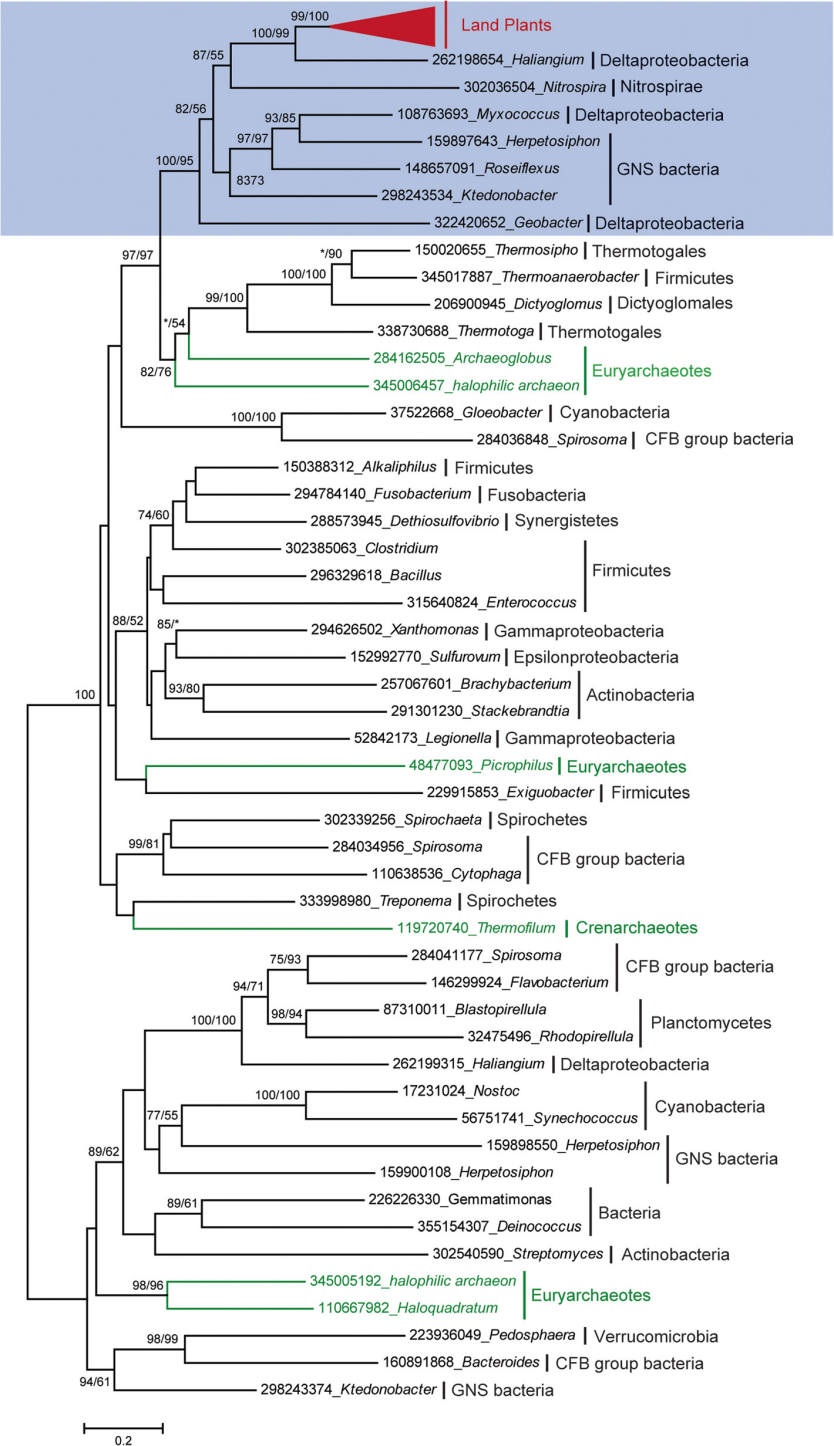
**Phylogenetic analyses of AEE proteins.** The numbers above the branches provide the bootstrap values for maximum likelihood and distance analyses. Asterisks indicate values lower than 50%. Blue shading indicates lineages in which land plant *AEE* genes evolved. Red, green and black branches indicate genes from land plants, archaea, and bacteria, respectively. All sequences were obtained from NCBI, except for those in green plants, and each protein is indicated by the GI numbers in NCBI and its genus.

To reveal the origin of the land plant *AEE* genes, we selected representative homologs from each taxonomic group of cellular organisms in the *nr* database to build a phylogenetic tree (Figure 2). Each of the proteins selected for phylogenetic analysis possessed an L-Ala-D/L-Glu_epimerase domain. Although the selected sequences cover most of the main taxonomy of the bacteria, the phylogenetic tree of the AEE proteins in our analysis is not congruent with the species phylogeny, suggesting extensive gene losses, HGT, and selection within bacteria. In the phylogenetic tree, all of the land plant *AEE* genes formed a single clade with high bootstrap support. The monophyly of the land plant *AEE* genes strongly suggests that they have a single origin and are derived from a unique gene that was already present in the ancestor of the land plants. In addition, we noted that the land plant *AEE* genes fell within the branch of bacterial genes showing high bootstrap support values in both maximum likelihood and distance analyses. The bacterial genes in this branch come from species of deltaproteobacteria, nitrospirae, and green non-sulfur (GNS) bacteria. In addition, we observed that the land plant AEE proteins showed the highest similarity to the bacterial genes in this clade; in particular, a motif in the C terminus is only conserved in land plants and these bacterial AEEs (Additional file 1). These findings illustrated that the land plant *AEE* genes originated from a single ancient HGT event from bacteria prior to the separation of land plant lineages.

HGT has been demonstrated to be one of the major forces driving the evolution of prokaryotes. Recently, accumulating data have indicated that this process has also occurred during the evolution of eukaryotic genomes [5]. However, in eukaryotes, another contributor to the accumulation of nuclear genes is intracellular gene transfer (IGT), which is gene transfer from the genomes of mitochondria or plastids to the nucleus [3]. It has been demonstrated that all mitochondrial genomes originated from an ancient endosymbiotic uptake of an alphaproteobacterium [24]. In addition, the chloroplast genome has been confirmed to have originated from the genome of an ancestor of extant cyanobacteria [25]. Thus, genes showing cyanobacterial and plastid-containing eukaryotic homologs as top hits were mostly considered plastid derived, while those with alphaproteobacterial and other eukaryotic homologs as top hits were considered to likely have been mitochondrion derived [5]. In our analysis, additional searches were performed to exclude the possibility of an IGT origin of land plant *AEE* genes. First, we searched the *nr* database, NCBI dbEST database and available eukaryotic genomic databases and found that there was no eukaryotic gene encoding an AEE other than those in land plants. Second, database searches revealed that no AEE protein was encoded by a mitochondrial or chloroplast gene. Third, although there were *AEE* genes found in both alphaproteobacteria and cyanobacteria, none of the *AEE* genes in these bacteria fell within the land plant branch in the phylogeny. Therefore, the scenario that land plant *AEE* genes originated through IGT requires too many independent gene loss events to seem likely. In addition, the hypothesis that this gene was present in the common ancestor of eukaryotes and only retained in land plants also requires numerous independent gene losses. Thus, under the assumption that the chance of the same gene being repeatedly transferred among different organismal groups is relatively low, the most parsimonious explanation is that the origin of land plant *AEE* genes was the result of an ancient HGT event from bacteria.

### Selective constraints on land plant *AEE* genes

Likelihood ratio tests of positive selection were applied using ML methods and the codon substitution models of Yang and his colleagues [26-28]. First, we compared models M0 and M3 to evaluate whether there were variations in the *d*_*N*_/*d*_*S*_ ratio among codon positions in the *AEE* genes of land plants (Additional file 2). Overall, the maximum likelihood estimate of the *d*_*N*_/*d*_*S*_ value for land plant *AEE* genes under model M0 was 0.1401, suggesting that relaxed purifying selection was the predominant force for the evolution of the *AEE* genes in land plants. Interestingly, the log-likelihood differences between models M3 and M0 were statistically significant (LRT=644.6478, *p* <0.01), illustrating that the overall level of selective constraint has fluctuated. Second, the LRTs employed to compare the fit of the data to model M2a vs. M1a and M8 vs. M7 were used to address whether positive selection promoted the divergence of this family in land plants. To our surprise, neither of these comparisons provided evidence of positive selection. This result revealed that the main constraint on the evolution of *AEE* genes in land plants was relaxed purifying selection following fixation after an HGT event. To compare the driving forces with the *AEE* genes in bacteria, 7 bacterial *AEE* genes that fell within the same branch as those in land plants were also selected to test selective constraints. The results revealed that purifying selection was the predominant force for the evolution of the *AEE* genes in bacteria, and no positive selection signature was found during their evolution in bacteria.

To test whether positive selection played a role in the fixation of this gene in the genome of the land plant ancestor following HGT, the improved branch-site model [29] was employed to detect positively selected amino acid sites. In this analysis, we used the branch of land plants as the foreground, while 7 bacterial genes that were present in the same branch as the land plant *AEE* genes in the phylogeny were used as the background. We found that the model that permitted a class of positively selected codons with *d*_*N*_*/d*_*S*_ >1 for the land plant branch was a significantly better fit to the data than the model in which this class of codon was restricted to *d*_*N*_*/d*_*S*_ =1 (Table 2). Because an LRT suggested that positive selection acted on the fixation of the *AEE* gene in the land plants, the Bayes empirical Bayes method was used to evaluate the positively selected sites and their posterior probabilities. A total of 34 codons were identified as showing a >50% posterior probability of a *d*_*N*_*/d*_*S*_ >1 along the land plant branch. Among these codons, 14 amino acid sites were identified as exhibiting a >95% posterior probability of positive selection, and 6 sites presented a value higher than 99%. For example, site 18 was found to be influenced by positive selection with a posterior probability of greater than 95%. In all of the selected bacterial *AEE* genes, this codon encodes a glycine (G), whereas it encodes a serine (S) in plants. However, site 37, which also shows a >95% posterior probability of positive selection, is quite divergent among the selected bacterial genes, while it is conserved and encodes a valine (V) in all of the land plant *AEE* genes. This alternation might be the result of positive selection at these sites and have contributed to the functional adaptation and fixation of this gene in land plants.

**Table 2:** Parameters of the branch-site models used for the detection of positive selection

**Model**	**1n *L***	**Parameters**
Null	−16116.4086	*p*_0_ = 0.6577, *p*_1_ = 0.1957, *p*_2*a*_ = 0.1130, *p*_2*b*_ = 0.0336
		Background: *ω*_0_ = 0.0838, *ω*_1_ = 1.0000, *ω*_2*a*_ = 0.0838, *ω*_2*b*_ = 1.0000
		Foreground: *ω*_0_ = 0.0838, *ω*_1_ = 1.0000, *ω*_2*a*_ = 1.0000, *ω*_2*b*_ = 1.0000
Alternative	−16102.3265^**^	*p*_0_ = 0.6537, *p*_1_ = 0.1911, *p*_2*a*_ = 0.1201, *p*_2*b*_ = 0.0351
		Background: *ω*_0_ = 0.0845, *ω*_1_ = 1.0000, *ω*_2*a*_ = 0.0845, *ω*_2*b*_ = 1.0000
		Foreground: *ω*_0_ = 0.0845, *ω*_1_ = 1.0000, *ω*_2*a*_ = 999.0000, *ω*_2*b*_ = 999.0000

## Discussion

The origin of land plants has played fundamental roles in the formation of modern terrestrial ecosystems [12]. Numerous lines of evidence have revealed that land plants evolved from water-based green algae. In the transmission from water to land, the ancestor of land plants is expected to have evolved genes with new functions to colonize the land. Among the mechanisms underlying the formation of these genes, HGT is one essential way to acquire new genetic material [30]. Recent investigations have revealed that HGT-derived genes play important roles in plant colonization of land, as some land plant genes that function in plant-specific activities, including plant defense, stress tolerance and the biosynthesis of plant polyamines and hormones, have been demonstrated to have been acquired through HGT [14]. In the present work, using integrated information on the taxonomic distribution, phylogenetic relationships and sequence similarity of the proteins possessing L-Ala-D/L-Glu_epimerase domains, we concluded that an ancient HGT event from bacteria contributed to the origin of *AEE* genes in land plants. The function of these genes in bacteria was demonstrated to be metabolism of the murein peptide of peptidoglycan [16,17]. Although no peptidoglycan has been found in land plants, the ubiquity of *AEE* genes in land plants and the evidence of their expression indicate that they are functional and may play important roles in the growth and development of plants. These genes are expected to exhibit other functions or have evolved new functions in land plants.

In this study, we also noted that all of the *AEE* genes include introns in their coding regions and that the positions and phases of these introns are quite conserved, illustrating that most of the introns were present in the ancestor of land plants. The vast majority of prokaryotic genes contain no introns, and the only introns that have been shown to be present in prokaryotic genes are self-splicing type II introns, which are functionally quite distinct from the spliceosome-dependent nuclear introns in eukaryotic genes [31]. Because no sequences showed highly similarity with these introns, it is currently unclear where they originated. However, it is conceivable that the introns in land plant *AEE* genes arose through insertions shortly after the HGT event and before the separation of land plant lineages.

It has been demonstrated that the phenotypic diversity of a gene family is controlled by selection as a function of evolutionary fitness [32]. A rigorous and clear signal of selection pressure in molecular evolution is a significantly higher nonsynonymous (*d*_*N*_; resulting in amino acid replacement) than synonymous (*d*_*S*_; silent) substitution rate. The ratio of the two rates, *d*_*N*_*/d*_*S*_, or *ω*, measures the quantity and direction of selective pressure on a protein, where *ω*≈1, *ω*<1, and *ω*>1, indicate neutral evolution, purifying selection, and positive selection, respectively [33]. Purifying selection is important for the evolution of a gene family because it can help the genes that belong to a family maintain their optimal function. However, positive selection is an important source of evolutionary innovation and is a major force underlying the adaptation of species to a new environment [34]. In our analysis, we found that the dominant driving force for *AEE* genes was purifying selection in both land plants and bacteria, which would contribute to functional stabilization. However, when we employed the bacterial genes as background, positive selection was found to contribute greatly to the evolution of land plant *AEE* genes.

In general, positive selection is thought to act on only a few amino acid sites and for a short evolutionary period [35]. Land plant *AEE* genes originated from bacteria through HGT as well as both the genomic and living conditions differ tremendously between bacteria and land plants. The functional adaptation of the *AEE* genes to the genomic and living environment of the ancestor of land plants was aided by positive selection. A successful HGT event leading to gene fixation results from providing a benefit to the host. Through positive selection, the *AEE* gene underwent complete functional innovation during a short evolutionary period in the ancestor of land plants. It is thought that if a transferred protein is not functional, neutral mutation will occur in the gene encoding it. The fate of the transferred gene will therefore be that it will be lost during evolution because of the accumulation of mutations. In addition to facilitating the adaptation of an organism to a particular niche, HGT can also provide a mechanism for genomic innovation and plasticity. After acquiring materials for innovation and adaptation to a new environment, positive selection acting on the transferred gene will modify its sequences to generate new functions. Thus, positive selection will reduce the chances of transferred gene losses caused by the accumulation of mutations.

## Conclusions

The gene encoding L-Ala-D/L-Glu epimerase (AEE) was found to be present in all of the available sequenced genomes of land plants, whereas homologs of this gene were not found in any other eukaryotic genome, including those of green and red algae. In this study, we performed extensive analyses of the taxonomic distribution and phylogeny of the AEE protein, which catalyzes the epimerization of L-Ala-D/L-Glu and other dipeptides and plays an important role in the metabolism of the murein peptide of peptidoglycan in bacteria. Our results revealed that the ancestor of land plants acquired the *AEE* gene from bacteria through an ancient HGT event. We also noted that rapid evolution and drastic sequence variation occurred during the initially short evolutionary period of the *AEE* gene in land plants following HGT. In addition to generating additional introns in the coding region of the gene, adaptive evolution via positive selection helped the *AEE* to undergo functional innovation and fixation in the genome of the land plant ancestor.

## Methods

### Sequence data sources

To identify the land plant genes encoding AEEs, BLASTP searches were performed in the Phytozome database [36] using the amino acid sequence of the *B. subtilis YkfB* gene [16] as a query. The CD-search tool in the Conserved Domain Database [37] of NCBI was used to predict the L-Ala-DL-Glu_epimerase domain (cd03319) for the obtained BLAST hits. The proteins that contained this conserved domain were defined as land plant AEEs. The new AEE sequences detected in land plants were used reiteratively to search the respective sequence database. EST searches for land plant *AEE* genes were performed using the BLASTN tool against the EST database of NCBI.

To identify the homologs of land plant *AEE* genes, BLAST searches against the non-redundant (*nr*) protein sequence database, NCBI EST database and available eukaryotic genome databases (Additional file 3) were performed using the land plant AEE protein sequences as queries. The obtained hits were further analyzed via an NCBI conserved domain search to confirm the presence of the L-Ala-D/L-Glu_epimerase domain in their protein structure. Protein sequences were sampled for further combined phylogenetic analysis from representative groups within each domain of life (bacteria, archaea, and eukaryotes) based on the BLASTP results.

### Multiple sequence alignment and phylogenetic tree reconstruction

All of the selected representative protein sequences were aligned using Clustal X [38]. The gaps and ambiguously aligned sites were removed manually. Phylogenetic analyses were performed using a maximum likelihood (ML) approach with PhyML version 3.0 [39] and a neighbor-joining (NJ) method using MEGA [40]. The ML phylogenetic analyses were conducted with the following parameters: JTT model, estimated proportion of invariable sites, 4 rate categories, estimated gamma distribution parameter, and optimized starting BIONJ tree. The JTT model was also employed for the construction of NJ trees. A total of 100 non-parametric bootstrap samplings were carried out to estimate the support level for each internal branch for both the ML and NJ trees. The branch lengths and topologies of all phylogenies were calculated with PhyML. Phylogenetic trees were visualized using the *explorer* program in MEGA.

### Detection of positive selection

A phylogenetically based maximum likelihood method was used to estimate the selective pressure acting on coding regions. The values of the *d*_*N*_*/d*_*S*_ ratio (or *ω*) for the land plant and selected bacterial *AEE* genes were calculated using the program *codeml* from PAML v4.4 [26]. The PAL2NAL program [41] was utilized for conversion of the protein sequence alignment into the corresponding codon-based nucleotide alignment, which, in turn, was input into the *codeml* program in PAML. Using the *codeml* program, we detected a variation in *ω* between sites by employing likelihood ratio tests (LRTs) of M0 vs. M3, M1a vs. M2a, and M7 vs. M8. The LRT for the M0 vs. M3 comparison was used to test the heterogeneity in *ω* between the codon sites, while the other two LRTs were used to detect the role of positive selection. For one LRT, twice the difference of the log likelihood of the two models was compared with chi-square (*χ*^*2*^) statistics, with degrees of freedom (DFs) equal to the difference in the number of parameters. In our analyses, the DFs were 3 for the M0/M3 test and 2 for the M1a/M2a and M7/M8 tests [28,42].

An improved branch-site model [29] was also used to detect the role of positive selection acting on the land plant *AEE* gene following HGT. For this analysis, we compared the null hypothesis (*ω* fixed to 1) with the alternative hypothesis (free *ω*) to test whether positive selection acted on the evolution of land plant *AEE* genes. A phylogenetic tree was generated using the land plant and bacterial *AEE* genes with the program PHYML. Here, only the bacterial genes falling within the same branch as the land plant genes were used. The land plant branch was used as the foreground, while the branch containing the genes from bacteria, the putative donors of the land plant *AEE* gene, was used as the background. The Bayes empirical Bayes procedure [27] in *codeml* was used to calculate the posterior probability that each site was subject to positive selection in the foreground branch.

## Competing interests

The authors declare that they have no competing interests.

## Authors’ contributions

ZY and CX designed all the analyses and wrote the manuscript. ZY, YW, QG, and EZ performed the analyses and participated in manuscript writing. YW, YZ, LZ, and YH contributed to the data analyses. All authors read and approved the final manuscript.

## Supplementary Material

Additional file 1 **Supplementary file 1.** The alignment of AEE sequences used for the phylogeny construction. The conserved motif was indicated by a box. Click here for file

Additional file 2 **Supplementary file 2.** The parameters of site-specific models. Click here for file

Additional file 3 **List of 50 eukaryotes whose complete genome sequences or ESTs were used in this study, in addition to NCBI *nr *database.** Click here for file
