# Supplementary material for: Evolution of land plant genes encoding L-Ala-D/L-Glu epimerases (AEEs) via horizontal gene transfer and positive selection
Source: BMC Plant Biol. 2013 Mar 1;13:34. doi: 10.1186/1471-2229-13-34 (PMC3605383; doi:10.1186/1471-2229-13-34)
Supplement: Additional file 1 — Supplementary file 1. The alignment of AEE sequences used for the phylogeny construction. The conserved motif was indicated by a box. [file 1471-2229-13-34-S1.doc]

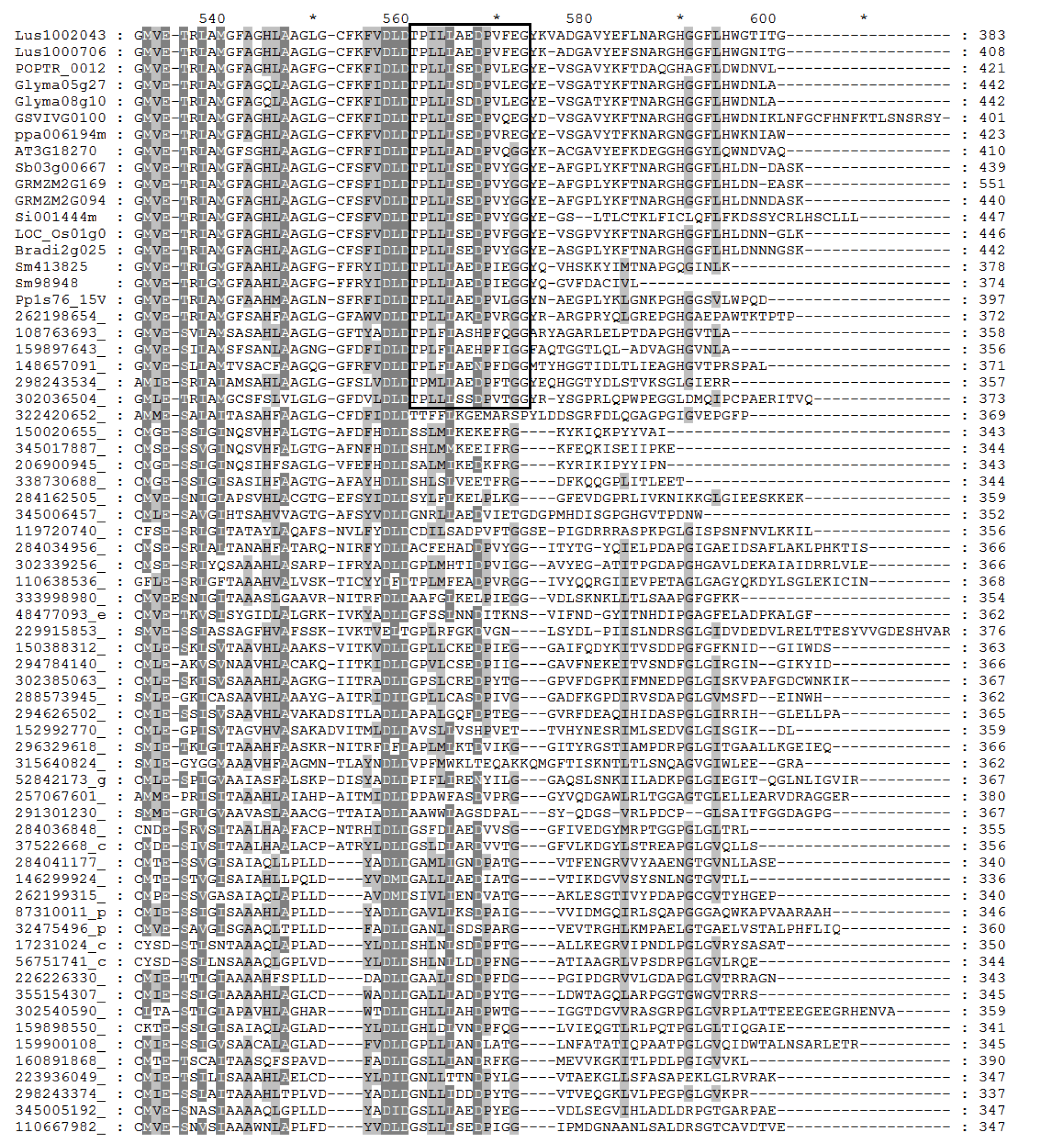


Supplementary file 1. The alignment of AEE sequences used for the phylogeny construction. The conserved motif was indicated by a box.
