# Supplementary material for: Evolution of land plant genes encoding L-Ala-D/L-Glu epimerases (AEEs) via horizontal gene transfer and positive selection
Source: BMC Plant Biol. 2013 Mar 1;13:34. doi: 10.1186/1471-2229-13-34 (PMC3605383; doi:10.1186/1471-2229-13-34)
Supplement: Additional file 2 — Supplementary file 2. The parameters of site-specific models. [file 1471-2229-13-34-S2.doc]

Supplementary file 2. The parameters of site-specific models

| Branch | Model | $\ln L$ | Parameters | PSS under NEB^a^ | PSS under BEB^b^ |
| --- | --- | --- | --- | --- | --- |
| Land Plants | M0 | -9918.8395 | $\omega=0.1401$ | - | - |
|  | M3 | -9596.5156^**^ | $p_{0}=0.4928,p_{1}=0.4127,p_{2}=0.0944$ $\omega_{0}=\text{0.0169,}\omega_{1}=\text{0.2299,}\omega_{2}=0.7447$ | NAN | NAN |
|  | M1a | -9738.7908 | $p_{0}=0.7544,p_{1}\text{=0.2456}$ $\omega_{0}=\text{0.0841,}\omega_{1}=1.0000$ | - | - |
|  | M2a | -9738.7908 | $p_{0}=0.7544,p_{1}=0.1590,p_{2}=0.0866$ $\omega_{0}=\text{0.0841,}\omega_{1}=\text{1.0000,}\omega_{2}=1.0000$ | NAN | NAN |
|  | M7 | -9598.3077 | $\beta(0.3998,1.8573)$ | - | - |
|  | M8 | -9596.8567 | $\beta(0.3998,1.8573)$ $p_{1}=\text{0.0165,}\omega\text{=1.3079}$ | 70R, 78E, 224R | 67V, 70R, 77R, 78E, 224R, 332C |
| Bacteria | M0 | -8046.8875 | $\omega=0.0063$ | - | - |
|  | M3 | -7866.9841^**^ | $p_{0}=0.3203,p_{1}=0.6797,p_{2}=0.0000$ $\omega_{0}=\text{0.0012,}\omega_{1}=\text{0.0119,}\omega_{2}=128.0398$ | NAN | NAN |
|  | M1a | -7927.7733 | $p_{0}=0.7329,p_{1}\text{=0.2672}$ $\omega_{0}=\text{0.0087,}\omega_{1}=1.0000$ | - | - |
|  | M2a | -7927.7733 | $p_{0}=0.7329,p_{1}=0.1620,p_{2}=0.1052$ $\omega_{0}=\text{0.0087,}\omega_{1}=\text{1.0000,}\omega_{2}=1.0000$ | NAN | NAN |
|  | M7 | -7847.8338 | $\beta(0.7855,59.0433)$ | - | - |
|  | M8 | -7847.8338 | $\beta(0.7852,58.9670)$ $p_{1}=\text{0.0001,}\omega\text{=32.4281}$ | NAN | NAN |

Note: a: positively selected sites under Naive Empirical Bayes (NEB) analysis; b: positively selected sites under Bayes Empirical Bayes (BEB) analysis.
