# Supplementary material for: Evolution of land plant genes encoding L-Ala-D/L-Glu epimerases (AEEs) via horizontal gene transfer and positive selection
Source: BMC Plant Biol. 2013 Mar 1;13:34. doi: 10.1186/1471-2229-13-34 (PMC3605383; doi:10.1186/1471-2229-13-34)
Supplement: Additional file 3 — List of 50 eukaryotes whose complete genome sequences or ESTs were used in this study, in addition to NCBI nr database. [file 1471-2229-13-34-S3.doc]

**Additional file 3: List of 50 eukaryotes whose complete genome sequences or ESTs were used in this study, in addition to NCBI *nr* database**

| **Species** | **Lineage** |
| --- | --- |
| *Acanthamoeba castellanii* | Amoebozoa |
| *Antonospora locustae* | Microsporidians |
| *Aureococcus anophagefferens* | Pelagophytes |
| *Batrachochytrium dendrobatidis* | Chytrids |
| *Bigelowiella natans* | Cercozoans |
| *Blastocystis hominis* | Stramenopiles |
| *Brachionus plicatilis* | Rotifers |
| *Capitella sp.* | Segmented Worms |
| *Capsaspora owczarzaki* | Opisthokonta |
| *Chlorella sp.* | Green Algae |
| *Chlorella vulgaris* | Green Algae |
| *Cyanidioschyzon merolae* | Red Algae |
| *Cyanophora paradoxa* | Glaucocystophyceae |
| *Daphnia pulex* | Crustaceans |
| *Dictyostelium purpureum* | Cellular Slime Molds |
| *Emiliania huxleyi* | Haptophytes |
| *Euglena gracilis* | Euglenoids |
| *Glaucocystis nostochinearum* | Glaucocystophyceae |
| *Guillardia theta* | Cryptomonads |
| *Hartmannella vermiformis* | Amoebozoa |
| *Heterocapsa triquetra* | Dinoflagellates |
| *Hyperamoeba sp.* | Amoebozoa |
| *Isochrysis galbana* | Haptophytes |
| *Karenia brevis* | Dinoflagellates |
| *Karlodinium micrum* | Dinoflagellates |
| *Lottia gigantea* | Gastropods |
| *Malawimonas jakobiformis* | Malawimonadidae |
| *Mastigamoeba balamuthi* | Amoebozoa |
| *Monosiga ovata* | Choanoflagellates |
| *Naegleria gruberi* | Heterolobosea |
| *Paracercomonas marina* | Cercozoans |
| *Pavlova lutheri* | Haptophytes |
| *Physarum polycephalum* | Plasmodial Slime Molds |
| *Phytophthora ramorum* | Oomycetes |
| *Phytophthora sojae* | Oomycetes |
| *Polysphondylium pallidum* | Cellular Slime Molds |
| *Polytomella parva* | Green Algae |
| *Porphyra yezoensis* | Red Algae |
| *Prototheca wickerhamii* | Green Algae |
| *Reclinomonas americana* | Jakobida |
| *Rhizopus oryzae* | Fungi |
| *Sawyeria marylandensis* | Heterolobosea |
| *Scenedesmus obliquus* | Green Algae |
| *Seculamonas ecuadoriensis* | Jakobida |
| *Sphaeroforma arctica* | Opisthokonta |
| *Spironucleus vortens* | Diplomonads |
| *Streblomastix strix* | Oxymonadida |
| *Thalassiosira pseudonana* | Diatoms |
| *Trimastix pyriformis* | Unclassified Eukaryotes |
